# Enhancing retrieval capacity of the predictive brain through dorsolateral prefrontal cortex intervention

**DOI:** 10.1093/cercor/bhaf005

**Published:** 2025-02-05

**Authors:** Laura Szücs-Bencze, Teodóra Vékony, Orsolya Pesthy, Krisztián Kocsis, Zsigmond Tamás Kincses, Nikoletta Szabó, Dezso Nemeth

**Affiliations:** Department of Neurology, Albert Szent-Györgyi Clinical Center, University of Szeged, Semmelweis utca 6, 6725 Szeged, Hungary; Centre de Recherche en Neurosciences de Lyon CRNL U1028 UMR5292, INSERM, CNRS, Université Claude Bernard Lyon 1, 95 Boulevard Pinel, 69500 Bron, France; Gran Canaria Cognitive Research Center, Department of Education and Psychology, University of Atlántico Medio, Ctra. de Quilmes, 37, 35017 Las Palmas de Gran Canaria, Spain; Doctoral School of Psychology, ELTE Eötvös Loránd University, Kazinczy utca 23-27, 1075 Budapest, Hungary; Institute of Psychology, ELTE Eötvös Loránd University, Izabella utca 46, 1064 Budapest, Hungary; Department of Radiology, Albert Szent-Györgyi Clinical Center, University of Szeged, Semmelweis utca 6, 6725 Szeged, Hungary; Department of Radiology, Albert Szent-Györgyi Clinical Center, University of Szeged, Semmelweis utca 6, 6725 Szeged, Hungary; Department of Neurology, Albert Szent-Györgyi Clinical Center, University of Szeged, Semmelweis utca 6, 6725 Szeged, Hungary; Centre de Recherche en Neurosciences de Lyon CRNL U1028 UMR5292, INSERM, CNRS, Université Claude Bernard Lyon 1, 95 Boulevard Pinel, 69500 Bron, France; Gran Canaria Cognitive Research Center, Department of Education and Psychology, University of Atlántico Medio, Ctra. de Quilmes, 37, 35017 Las Palmas de Gran Canaria, Spain; BML-NAP Research Group, Institute of Psychology, Eötvös Loránd University and Institute of Cognitive Neuroscience and Psychology, HUN-REN Research Centre for Natural Sciences, Damjanich utca 41, 1072 Budapest, Hungary

**Keywords:** alternating serial reaction time task, dorsolateral prefrontal cortex, predictive processes, retrieval, statistical learning, transcranial magnetic stimulation

## Abstract

Extracting spatial or temporal patterns across experiences is essential for skill acquisition and predictive processes. The prefrontal cortex plays a central role in regulating competitive cognitive systems, with a particular influence on executive functions, often opposing statistical learning. This regulatory function may account for observed improvements in the acquisition and consolidation of statistical regularities following inhibition of the dorsolateral prefrontal cortex via repetitive transcranial magnetic stimulation. However, whether access to previously acquired statistical knowledge can similarly benefit from dorsolateral prefrontal cortex inhibition remains unclear. This preregistered study investigated the dorsolateral prefrontal cortex’s role in retrieving pre-existing statistical knowledge of temporal regularities. Healthy human participants engaged in an implicit probabilistic sequence learning task followed by a 24-h consolidation period. Before retesting, they received either 1 Hz repetitive transcranial magnetic stimulation or sham stimulation over the left, right, or bilateral dorsolateral prefrontal cortex for 10 min. We observed that retrieval of statistical regularities was enhanced in the Bilateral dorsolateral prefrontal cortex group compared to the Sham group. Our findings suggest that dorsolateral prefrontal cortex inhibition may facilitate access to statistical knowledge, particularly when interhemispheric compensatory mechanisms are limited. These insights advance our understanding of the dynamic neural background of statistical learning and may inform strategies for cognitive enhancement.

## Introduction

In daily life, different tasks demand distinct cognitive processes, often placing the top–down executive control system and the habitual system in competition for the same mental resources (Poldrack and Packard 2003; Borragán et al. 2016; Smalle et al. 2022). Statistical learning, defined as the incidental detection of regularities based on probabilities (Schapiro et al. 2012), aligns closely with habitual behavior, as both occur without conscious awareness (Obeid et al. 2016; Arciuli 2017). We therefore consider statistical learning a component of the habitual system (Horváth et al. 2022). In managing the competition between these two cognitive systems, the prefrontal cortex (PFC) plays a crucial role as a top–down controller. Numerous findings indicate that PFC-dependent executive functions and working memory often operate antagonistically to statistical learning (Virag et al. 2015; Pedraza et al. 2024), as suppressing PFC activity—via interventions like hypnosis (Nemeth et al. 2013), transcranial magnetic stimulation (TMS) (Ambrus et al. 2020; Smalle et al. 2022), or cognitive overload (Smalle et al. 2022)—can enhance statistical learning performance. Furthermore, neuroimaging studies suggest that a reduction in PFC engagement can favor habitual, bottom–up learning mechanisms (Tóth et al. 2017; Park et al. 2022), consistent with the notion of the competitive systems framework. Given these insights, a critical question arises: Does inhibiting the DLPFC shift cognitive balance toward the habitual system during statistical retrieval, thereby enhancing access to pre-existing statistical knowledge? Although the PFC’s involvement in predictive model formation is well documented, the specific mechanisms by which it facilitates or hinders access to statistical knowledge remain less understood.

Beyond moderating executive function engagement, the PFC may influence statistical learning by modulating activity in memory-related regions, notably the hippocampus. Both episodic memory and statistical learning rely on the hippocampus (Schapiro et al. 2014; Schapiro et al. 2016; Sherman et al. 2024a, 2024b) and these distinct memory types, at times, compete within this shared anatomical framework (Sherman and Turk-Browne 2020). The PFC is known to modulate hippocampal activity during memory retrieval (Benoit et al. 2015; Woodcock et al. 2015; Oehrn et al. 2018), thus potentially influencing the balance between episodic and statistical memory processing. Supporting this role, a neuroimaging study demonstrated that the structural connectivity between the hippocampus and dorsolateral PFC (DLPFC) predicts statistical learning performance, linking the DLPFC’s influence to predictive processing and the formation of statistical models (Bennett et al. 2011). Does the DLPFC influence the dynamics between the executive control system and the habitual system, or does it more directly mediate the competition between statistical learning and episodic memory within the hippocampus?

Noninvasive brain stimulation (NIBS) techniques, such as TMS, allow for precise testing of causal relationships between brain regions and cognitive functions. Previous work on DLPFC stimulation and statistical learning has yielded mixed results (Szücs-Bencze et al. 2023). Early studies reported reduced statistical learning following DLPFC stimulation (Pascual et al. 1996; Robertson et al. 2001), while later studies found improved statistical learning performance when the DLPFC was disrupted via TMS (Galea et al. 2010; Ambrus et al. 2020). In particular, Smalle et al. (2017, 2022) showed enhanced acquisition of linguistic regularities following DLPFC inhibition. Nonetheless, some studies found no significant effect of DLPFC stimulation on statistical learning (Wilkinson et al. 2010; Gann et al. 2021). The discrepancy could stem from differences in stimulation timing and protocol. Studies showing decreased learning applied either high-frequency repetitive TMS (rTMS), which is likely increased excitability (Pascual et al. 1996), or inhibitory TMS protocols applied before the learning phase (Robertson et al. 2001; Wilkinson et al. 2010; Gann et al. 2021). Conversely, studies reporting enhanced learning used inhibitory TMS protocol timed after the learning phase, promoting consolidation and subsequent knowledge retention (Galea et al. 2010; Tunovic et al. 2014; Ambrus et al. 2020). Given these findings, it is reasonable to question whether DLPFC inhibition affects retrieval processes similarly to learning. Beyond the scope of learning and acquisition, the application of probabilistic knowledge for predictive processes is a continuous necessity in daily life, requiring consistent access and retrieval. Could disrupting DLPFC activity shift mental resources away from goal-directed behavior, thus enhancing the retrieval of habitual processes like statistical learning?

In order to fill this gap, our study aimed to investigate the effect of inhibitory DLPFC stimulation on the retrieval of pre-existing knowledge of statistical regularities. Brodmann 9 was selected for targeting due to its established involvement in statistical learning and predictive processes (Galea et al. 2010; Ambrus et al. 2020; Smalle et al. 2022). The comprehensive mapping of the role of DLPFC was taken by targeting the left, right, and bilateral DLPFC in separate groups with low-frequency rTMS. We applied bilateral stimulation, which is a unique and surprisingly rarely used method in cognitive neuroscience research; however, this design is assumed to minimize compensatory mechanisms by the nonstimulated hemisphere. Moreover, as recent studies indicate that it is unjustified to determine TMS intensity based on the motor threshold when stimulating nonmotor cortical areas (Wassermann et al. 1992; Antal et al. 2004; Turi et al. 2022), we adopted a uniform intensity setting, similar to Ambrus et al. (2020). The fixed intensity was determined by simulating the electric field in the brain, using SimNIBS 4 (Thielscher et al. 2015). Statistical learning was measured using the Alternating Serial Reaction Time (ASRT) task (Howard et al. 2004), which reflects real-world statistical learning processes with high reliability (Farkas et al. 2023). To validate the specificity of rTMS effects on the retrieval of statistical knowledge, we used the Paired Associate Learning Task (PALT) as a control memory task, assessing declarative/episodic learning and recall. After participants learned on the tasks, a 24-h retention period ensued. Subsequently, prior to retesting (retrieval phase), participants received inhibitory stimulation in the form of 1 Hz rTMS, or sham stimulation for 10 min. We have outlined three potential hypotheses regarding the effects of DLPFC inhibition on statistical retrieval and episodic recall. (i) If DLPFC inhibition precludes access to long-term memory representation and cognitive models, we expect to observe a decrease in both statistical and episodic retrieval performance. (ii) If DLPFC inhibition weakens cognitive control, we anticipate that participants will show enhanced retrieval of statistical knowledge alongside decreased episodic recall. (iii) Lastly, if DLPFC inhibition does not affect the retrieval of previously learned statistical models, it would indicate that implicit statistical learning is an automatic and robust process, resistant to modulation by rTMS, particularly after a lengthy consolidation period. Additionally, based on previous findings (Ambrus et al. 2020), we expect that bilateral DLPFC stimulation has the greatest potential to enhance retrieval outcomes. This would prevent any compensatory mechanisms between the hemispheres that might occur when only one side of the DLPFC is stimulated.

## Materials and methods

### Participants

One hundred and four healthy adult volunteers were enrolled in this preregistered study (https://osf.io/jzubg). Two participants were excluded due to the disclosed history of neurological or psychiatric disorders, and one participant did not complete the study. Thus, the final sample consisted of 101 participants, all with normal or corrected-to-normal vision and no contraindications for TMS (pacemaker, history of major surgery, history of neurological or psychiatric disease, metal implant, pregnancy). None of the participants withdrew from the participation owing to TMS discomfort. At the start of the first session, after completing the TMS contraindication questionnaire, participants were randomly assigned to one of four groups: Left DLPFC, Right DLPFC, Bilateral DLPFC, or Sham, with 25, 26, 25, and 25 participants in each group, respectively (see Table 1 for descriptive statistics). Written informed consent was obtained from each subject. All study procedures were approved by the Regional Scientific and Research Ethics Committee of Albert Szent-Györgyi Clinical Center, University of Szeged (approval ID: 166/2020-SZTE RKEB), which complied with the Declaration of Helsinki’s ethical guidelines.

**Table 1:** Descriptive statistics of the four experimental groups.

	Group			
	Left DLPFC	Right DLPFC	Bilateral DLPFC	Sham
Gender (f/m)	14/11	18/8	13/12	17/8
Age (years)	23.76 ± 5.15	26.11 ± 7.26	22.40 ± 4.27	25.88 ± 6.02
Years of education	15.40 ± 2.70	15.73 ± 2.82	14.40 ± 2.50	16.08 ± 3.53
Handedness (r/l/a)	24/1/0	23/1/2	21/4/0	21/2/2
Counting Span Task	4.41 ± 0.62	4.28 ± 0.52	3.96 ± 0.76	3.98 ± 0.76

*Note*. Mean and SD values for age, years of education, and Counting Span Task are presented. For gender (f = female, m = male) and handedness (r = right, l = left, a = ambidexter), case numbers are presented.

### ASRT task

**Fig. 1:**
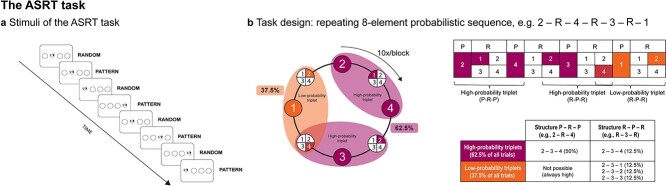
The ASRT task. a) Pattern elements alternate with random elements. b) An 8-element probabilistic sequence repeats 10 times during a block. Due to this sequence structure, some runs of three successive stimuli appeared with higher probability (high-probability triplets) than others (low-probability triplets). Each trial was categorized as the last element of high- or low-probability triplets. The RT difference between the two trial types indicates implicit statistical learning.

**Fig. 2:**
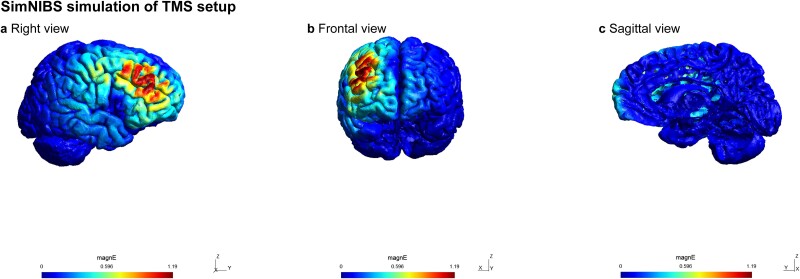
SimNIBS simulation of TMS setup. Demonstration of the spread of electrical field using SimNIBS 4 (“Earnie” head mash) when positioning the TMS coil over the right DLPFC (F4). Results are similar for the left DLPFC (F3) stimulation. SimNIBS did not allow us to simulate sequential bilateral stimulation. The electric field is represented as volts per meter (V/m).

Statistical learning was assessed via the ASRT task (Howard et al. 2004). The task was run in the E-Prime 3.02 software environment. Participants were presented with a stimulus (depicting a dog’s head) appearing in one of four empty circles arranged horizontally on the screen (see Fig. 1a). Their task was to press the corresponding keys (Z, C, B, or M on a QWERTY keyboard) swiftly and accurately. The participants were instructed to use their middle and index fingers of the left hand to press the Z and C keys and the same fingers of the right hand to press the B and M keys, respectively. The stimulus remained on the screen until the subject pressed the correct key, after which the next stimulus appeared 120 ms later (response-to-stimulus interval). Unbeknownst to the participants, the stimuli followed a probabilistic eight-element sequence, where pattern and random elements alternated (eg 2r4r3r1r, where numbers 1 to 4 denoted target locations from left to right, and “r” represented a randomly selected position of the four possible ones) (see Fig. 1b). The task comprised 25 blocks, each with 80 trials, repeating the eight-element sequences 10 times within each block. Due to the alternating pattern, the ASRT task provided the occurrence of certain sets of three consecutive stimuli (referred to as triplets) with varying probabilities. In high-probability triplets, the third element could be predicted based on the first element with higher probability (constituting 62.5% of all trials) compared to low-probability triplets, where the probability of the prediction of the third element from the first one was lower (constituting 37.5% of all trials) (see Fig. 2b). We categorized each trial in a sliding window manner based on whether it represented the third element of a high-probability or low-probability triplet. Statistical learning was defined as the reaction time (RT) difference between trials that were the third element of a high-probability triplet or a low-probability triplet. Besides, participants generally become faster on the task irrespective of triplet types, indicating general visuomotor performance.

### Control memory task

The PALT (Nagy et al. 2013) measuring declarative/episodic learning was utilized as a control memory task. The PALT was run using E-Prime 3.0 software. In the Learning session, participants were presented with 23 pairs of images. The images were presented side by side on the computer screen and depicted schematic drawings, with each image containing one object and one animal. After naming both pictures in a pair, the experimenter pressed a button to proceed to the next pair. During the Retrieval session, participants were shown 32 pairs of images and had to indicate whether (i) the two pictures were presented in the Learning session and, if yes, (ii) they were presented together or with another pair. The 32 pairs of images of the Retrieval session matched to 4 conditions, with 8 pairs per condition: (i) both pictures were presented together in the Learning session (Old–Old original), (ii) both pictures were presented in the Learning session but were paired with different images (Old–Old rearranged), (iii) one picture was presented in the Learning session while the other was not (Old–New or New–Old), and (iv) neither picture was presented in the Learning session (New–New). After selecting an answer, the experimenter recorded it by pressing the corresponding button (1 to 5), prompting the next pair of images to appear. A 500 ms fixation cross was displayed between each stimulus presentation.

### TMS protocol

**Fig. 3:**
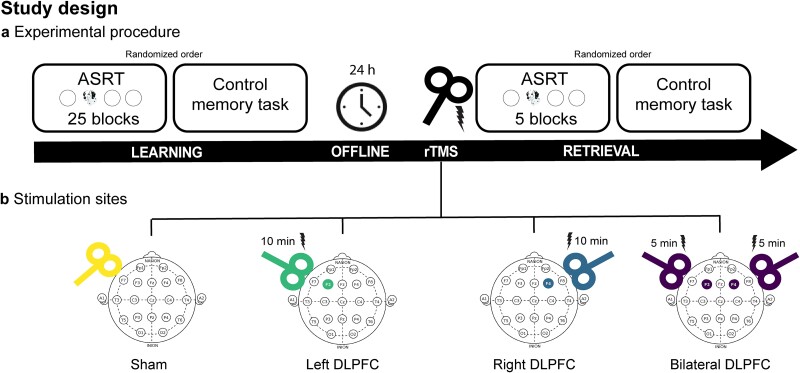
Study design. a) The study spanned two experimental days. On the first day, participants practiced the ASRT task through 25 learning blocks and performed the learning phase of the PALT; then, a 24-h offline period ensued. On the second day, participants received 1 Hz rTMS for 10 min. Immediately after rTMS administration, participants performed 5 blocks of the ASRT task and the recall phase of the PALT. The order of the two tasks was counterbalanced between participants on both days. b) Stimulation sites for the four groups: The coil was tilted by 90° in the Sham group, F3 was stimulated for 10 min in the left DLPFC group, F4 was stimulated for 10 min in the Right DLPFC group, and F3 and F4 were sequentially stimulated for 5 min each in the Bilateral group.

TMS stimulation was administered through a Magstim Rapid2 Stimulator equipped with a D702 70 mm figure-of-eight coil (The Magstim Company Ltd, Whitland, Wales, UK). Magnetic pulses were delivered at 1 Hz for 10 min, resulting in a total of 600 pulses. To determine our TMS setup, we used SimNIBS 4 (Thielscher et al. 2015). We demonstrate the results of the right DLPFC stimulation in Fig. 2 as similar results were found in the case of the left DLPFC stimulation. The stimulation intensity was uniformly set for all participants at 55% of the maximum stimulator output (MSO). We opted against the traditional motor threshold–based intensity setting as evidence indicated that this approach is inappropriate for stimulating regions outside the motor cortex (Wassermann et al. 1992; Antal et al. 2004; Turi et al. 2022). For instance, motor thresholds can vary significantly even among different upper extremity muscles (Wassermann et al. 1992). Moreover, there is no correlation between motor threshold and TMS-induced effects in other cortical areas, such as the induction of phosphenes in the visual cortex (Antal et al. 2004). These findings suggest that using motor threshold as a basis for determining stimulation intensity lacks clear scientific justification (Turi et al. 2022). Additionally, with this uniform intensity setting, TMS successfully modulated statistical learning in a prior study (Ambrus et al. 2020). TMS coil positioning followed the international 10–20 electroencephalography (EEG) system using an EEG cap—this method can be used with 90% accuracy to position the coil over the targeted area (Herwig et al. 2003). The center of the coil was placed at the location of the F3 electrode for left DLPFC stimulation and at F4 for right DLPFC stimulation (Brodmann 9) throughout the entire stimulation period. In the case of bilateral DLPFC stimulation, the coil was placed at the F3 location for the first half of the stimulation (5 min, 300 pulses) and then moved to F4 for the second half (see Fig. 3b). The order of stimulation for the hemispheres was counterbalanced across participants in the Bilateral group. For sham stimulation, the coil was tilted 90° away from the skull; thus, the participants could hear the noise made by the machine, but it had no effect on the brain functioning.

### Procedure

The study spanned two experimental days, during which participants engaged in tasks in a well-lit, quiet environment. The initial day involved participants performing the ASRT task across 25 blocks, lasting approximately 25 to 35 min, to acquire the 8-element probabilistic sequence, and the learning phase of the PALT, lasting approximately 10 min (Learning session). In the case of the ASRT task, the participants were unaware of the learning nature of the task. Additionally, on the first day, participants completed the Counting Span task to ensure that the four experimental groups did not differ in baseline cognitive functions (see Table 1). After a 24-h offline period, the second day involved the administration of rTMS and the retest of participants’ statistical and declarative knowledge (Retrieval session). The rTMS procedure, lasting 10 min, was immediately followed by the ASRT task comprising 5 blocks using the same alternating sequence practiced on the previous day or the recall phase of the PALT. The order of the statistical and declarative learning tasks was counterbalanced between and within participants in the two sessions (see Fig. 3a).

### Statistical analysis

#### ASRT

Trills (such as 1–2–1) and repetitions (such as 1–1–1) were omitted from the analysis because subjects might exhibit inherent response patterns for these trial types (Soetens et al. 2004). Additionally, trials with RTs below 100 ms and those exceeding three SDs above the mean RT were excluded, as they are unlikely to represent valid reactions. Trials with incorrect responses (misses) were also removed.

Statistical analysis was performed in R. Linear mixed models (LMMs) were fitted on block-wised mean RT data with the *mixed* function of the *afex* package, separately for the Learning Session and Retrieval Session. Trial Type (high- vs. low-probability), Group (Left, Right, Bilateral, Sham) and Block (Learning Session: 1 to 25; Retrieval Session: 26 to 30) were included as fixed factors. Subject factor was included as a random intercept, as well as by-participant correlated slopes for the Block factor. To assess the significant factors influencing the model’s quality, we conducted a likelihood ratio test, which pertained to both random and fixed effects, by utilizing the *anova* function in R. This test compared the likelihoods, as indicated by the Akaike information criteria (AIC) in LMMs, between two or more models. The model with the lowest AIC was considered the best model with the highest information gain (Bozdogan 1987). Estimated marginal means were computed with the *emmeans* R package. An alpha level of 0.05 was applied to all analyses. If necessary, Bonferroni correction was performed on post hoc paired comparisons.

#### PALT

Three learning indices could be distinguished based on the answers of the participants. The item memory index was calculated by subtracting the ratio of incorrect Old–Old responses to New–New pairs (false alarm) from the ratio of responses indicating recognition of Old–Old rearranged pairs (hit rate). The association learning index was quantified by subtracting the ratio of responses indicating recognition of rearranged Old–Old responses (hit rate) from the ratio of responses indicating recognition of original Old–Old responses (hit rate). Finally, the recollection index was defined by subtracting the ratio of incorrect Old–Old original responses to Old–Old rearranged pairs (false alarm) from the ratio of responses indicating recognition of Old–Old original pairs (hit rate). To compare the three PALT learning indices between the four groups, one-way analyses of variance (ANOVAs) were conducted.

## Results

### Comparable statistical learning performance in the four groups in the learning session

**Fig. 4:**
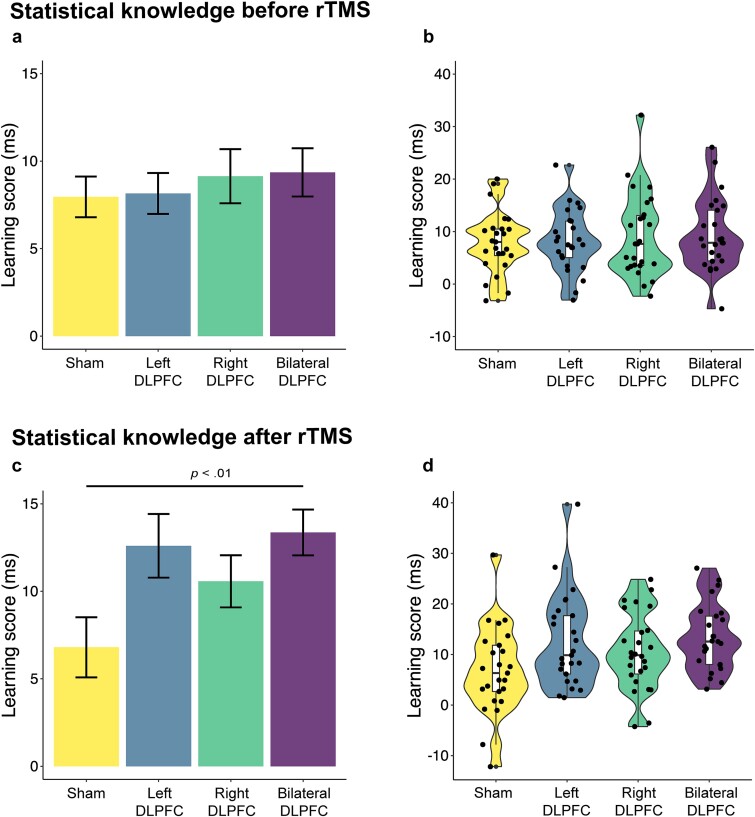
Statistical knowledge before and after rTMS. a) The *y* axis shows the mean statistical learning scores (RT difference between high- and low-probability trials in ms) of the four groups of the learning session. The DLPFC groups did not differ from the sham group in statistical learning. Error bars represent standard error. b) Individual statistical learning scores in the four groups. c) The *y* axis shows the mean statistical learning scores (RT difference between high- and low-probability trials in ms) of the four groups of the retrieval session. The Bilateral DLPFC group outperformed the Sham group in the retrieval of statistical knowledge (*P* < 0.01). Error bars represent standard error. d) Individual statistical learning scores in the four groups.

The best model included Trial Type, Block, and Group as fixed factors, where Block was also added as a by-participant random slope factor (see Supplementary Tables 1 and 2). A main effect of Trial Type was found, with high-probability trials showing faster RTs than low-probability trials, thus statistical learning occurred among all participants [*F*(1, 4840) = 278.76, *P* < 0.001]. The interaction between Trial Type and Block revealed a progressive improvement in statistical learning with gradually increasing differences between high- and low-probability triplets [*F*(1, 4840) = 33.62, *P* < 0.001]. However, no evidence was found to suggest a performance difference in statistical learning between the four groups, as indicated by the lack of significant interaction between Group and Trial Type [*F*(3, 4840) = 0.45, *P* = 0.714] and in the progression of statistical learning across blocks [*F*(3, 4840) = 0.32, *P* = 0.814] prior to stimulation (see Fig. 4a and b). The main effect of Block revealed decreasing RTs throughout the task, indicating the gradual improvement of visuomotor performance [*F*(1, 97) = 321.79, *P* < 0.001]. Nevertheless, no evidence was found to suggest a difference in visuomotor performance between groups, as neither the main effect of Group [*F*(3, 97) = 0.05, *P* = 0.987] nor the interaction between Group and Block [*F*(3, 97) = 2.09, *P* = 0.106] reached significance.

### Enhanced retrieval capacity of statistical knowledge after bilateral DLPFC inhibition in the retrieval session

The best model regarding the Retrieval session included Trial Type and Group as fixed factors, as well as their interaction, and Block as a by-participant random slope factor (see Supplementary Tables 3 and 4). Intact statistical learning was evidenced by the main effect of Trial Type, with greater speed for high-probability triplets compared to low-probability ones [*F*(1, 804) = 199.10, *P* < 0.001]. The lack of a main effect of Group indicated no evidence of a difference in visuomotor performance between the four groups [*F*(3, 97) = 0.41, *P* = 0.743]. Nonetheless, the interaction between Group and Trial Type revealed a difference in overall statistical learning between the four groups [*F*(3, 804) = 3.62, *P* = 0.013]. According to post hoc Welch’s *t*-tests, two stimulation groups demonstrated better statistical learning performance compared to the Sham group: the Bilateral DLPFC group [*t*(44.835) = 3.04, *P* < 0.01] and the Left DLPFC group [*t*(47.839) = 2.32, *P* < 0.05], which remained significant only in the Bilateral DLPFC group after correction for multiple comparisons (see Fig. 4c and d). Mean RTs for high- and low-probability trials across all blocks (1 to 30) are presented separately for each group in Supplementary Fig. 1.

### Intact recall capacity on the control memory task in the retrieval session

The four groups were found to be comparable in declarative performance, with no evidence of differences in the item memory index [*F*(3, 97) = 0.852, *η^2^p* = 0.026, *P* = 0.469], association learning index [*F*(3, 97) = 0.506, *η^2^p* = 0.015, *P* = 0.679], or recollection index [*F*(3, 97) = 0.347, *η^2^p* = 0.011, *P* = 0.791].

## Discussion

In this study, we investigated a pivotal aspect of predictive processing: the function of the DLPFC in retrieving predictive models. Here, we aimed to fill this gap by administering low-frequency rTMS over the left, right, and bilateral DLPFC before retesting participants’ statistical knowledge acquired 24 h prior. Our results indicate that disrupting the DLPFC enhances retrieval capacity, particularly if we stimulate both hemispheres. Since general visuomotor performance (speed regardless of trial probability) and the control memory task remained unaffected by rTMS intervention, this boosting effect is presumed to be specific to statistical learning. These findings suggest that less DLPFC involvement not only aids in the acquisition, as found by Ambrus et al. (2020), but also the retrieval of temporally distributed predictable patterns. Our findings align with previous research demonstrating improved statistical learning following DLPFC suppression by rTMS. Smalle et al. (2017, 2022) applied continuous theta burst stimulation (cTBS), and they showed increased learning of linguistic sequences after cTBS over the left DLPFC. Galea et al. (2010) stimulated the left and right DLPFC in separate groups with cTBS immediately after training on a nonlinguistic motor sequence learning task. They found improved learning capacity 8 h later in both groups compared to control, with greater improvement after right DLPFC stimulation than after left DLPFC. Similarly, applying cTBS over the right DLPFC after training led to offline improvement in nonlinguistic statistical learning, whereas its facilitatory counterpart did not have a boosting effect (Tunovic et al. 2014). These findings confirm the notion that the PFC plays a crucial role in acquiring statistical regularities and, now, supplemented by our findings, also in the retrieval processes.

Studies demonstrating increased statistical learning capacity on cognitive depletion through DLPFC inhibition interpret their findings within the framework of competitive cognitive systems (Galea et al. 2010; Borragán et al. 2016; Smalle et al. 2017; Ambrus et al. 2020; Smalle et al. 2022). Within this framework, the executive control system, which relies heavily on the PFC, competes with the habitual system for the same mental resources (Poldrack and Packard 2003; Gillan et al. 2011). The PFC is believed to promote goal-directed processes such as cognitive control and executive functions while impeding habitual, associative learning processes (Janacsek et al. 2012; Juhasz et al. 2019; Smalle and Möttönen 2023). Therefore, reduced involvement of the DLPFC allows for more cognitive resources to be available for statistical learning mechanisms.

Competition may also occur on another level—not between overarching cognitive systems but directly between episodic memory and statistical learning, which both share the hippocampal circuitry. Several studies have shown that the PFC exerts inhibitory control over the hippocampus, influencing memory processes, including retrieval (Benoit et al. 2015; Woodcock et al. 2015; Oehrn et al. 2018). However, if we assume that the PFC functions as a top–down controller shifting between these two memory types, DLPFC inhibition should theoretically impact both statistical and episodic retrieval. Thus, why did we not find any effect of DLPFC inhibition on retrieval in the control memory task? Research suggests that the dorsal stream of the PFC is responsible for executive control during episodic retrieval, with the DLPFC’s engagement being strategy-dependent—higher when retrieval strategies are applied (Kim 2010; Manenti et al. 2010). In the present study, the episodic learning process was only partially driven by conscious control, as learning occurred incidentally and only retrieval was intentional. This suggests that the declarative learning task did not engage DLPFC-dependent top–down processes sufficiently. Moreover, within the hippocampus, two anatomically distinct pathways support learning: the monosynaptic pathway, which underlies statistical learning, and the trisynaptic pathway, which supports episodic memory (Schapiro et al. 2017; Sherman et al. 2024b). It is possible that the PFC’s influence in the hippocampus diverges and selectively affects the monosynaptic pathway associated with statistical learning. Future studies should employ more strategically demanding episodic tasks to test for potential competition between these memory types.

If we set aside the competition model, an alternative explanation for our findings could involve the strengthening of the frontostriatal network, rather than a direct competition between cognitive systems. It is essential to consider that cognitive functions are supported by extensive brain networks rather than isolated brain areas, meaning that TMS and similar NIBS techniques do not selectively modulate specific brain regions in isolation (Beynel et al. 2020; Bergmann and Hartwigsen 2021). In the case of statistical learning, the neural foundation is the functional connection between frontal regions and the basal ganglia, collectively known as the frontostriatal network (Naismith et al. 2010; Reber 2013; Janacsek et al. 2020). Studies indicate that reduced PFC engagement and decreased connectivity with other regions can favor statistical learning outcomes (Park et al. 2022; Tóth et al. 2017), suggesting that TMS-induced DLPFC inhibition may optimize conditions within this network, leading to improved access to statistical knowledge. This noncompetitive model suggests that the observed effect may arise not from direct competition between cognitive systems but rather from enhanced support within this critical network. Future research is warranted to combine functional magnetic resonance imaging and NIBS to explore further how DLPFC inhibition influences the frontostriatal network’s dynamics and how these changes interact with statistical learning and retrieval.

However, apparent contradictions arise regarding lateralization. In our research, the retrieval performance of the Left and Right DLPFC groups was similar to the retrieval capacity of the Bilateral DLPFC group, yet it did not surpass that of the Sham group, even though other studies found unilateral stimulation to be effective (Tunovic et al. 2014; Smalle et al. 2017). One possible reason for this discrepancy could be the different stimulation protocols, as rTMS and TBS could exert different effects on statistical learning (Verwey et al. 2022). Furthermore, the complexity of statistical information present in the input could be a significant factor. In our study, right DLPFC inhibition was the least effective among the three active stimulation conditions in modulating retrieval capacity, whereas other studies found right hemisphere stimulation to be effective (Tunovic et al. 2014), even more effective than stimulation of the left DLPFC (Galea et al. 2010). However, those studies used deterministic sequences, which, unlike the probabilistic sequences we utilized, have a much simpler structure and are less effective in accurately reflecting real-life statistical learning processes. In our study, the Left DLPFC group, similar to the Bilateral DLPFC group, achieved significantly better retrieval compared to the Sham group, but this performance difference disappeared after correction for multiple comparisons. In another study, where statistical learning of linguistic sequences was investigated, inhibitory stimulation of the left DLPFC also led to better learning. These findings suggest that more complex sequences with higher ecological validity are likely to rely more on the left hemisphere. Further studies are needed to support the lateralization of statistical learning and retrieval in the PFC.

Our finding of the superiority of bilateral stimulation over unilateral stimulation is highly consistent with that of a previous study (Ambrus et al. 2020). They administered 1 Hz rTMS to the DLPFC bilaterally between the learning blocks and observed subsequently enhanced learning capacity of probabilistic regularities compared to the Sham group. The authors suggested that sequential bilateral stimulation, where the same rTMS protocol is successively applied to both hemispheres, may prevent potential interhemispheric compensatory mechanisms. In our study, the statistical knowledge following left and right DLPFC stimulation approached that of bilateral stimulation, albeit not to the extent that it surpassed the performance of the Sham group. This is likely due to the unstimulated hemisphere compensating for the other’s function, thereby neutralizing the stimulation effect. Another important consideration is that studies where only one hemisphere was stimulated typically involve one-handed versions of motor learning tasks (Szücs-Bencze et al. 2023). For studies using two-handed tasks, the bilateral approach is generally more advisable.

In conclusion, we demonstrated the functional role of DLPFC in retrieving previously acquired statistical knowledge, a key component in predictive coding and processing. The most significant enhancement in retrieval capacity occurred with bilateral inhibition, suggesting that suppressing the DLPFC is particularly beneficial when interhemispheric compensatory mechanisms are limited. These findings enrich our understanding of the PFC’s role in predictive processing and possible contributions to switching between competing cognitive systems and have implications for cognitive enhancement strategies.

## Supplementary Material

TMS_Szeged_Supplementary_material_final_bhaf005
