# Supplementary material for: Enhancing retrieval capacity of the predictive brain through dorsolateral prefrontal cortex intervention
Source: Cereb Cortex. 2025 Feb 5;35(2):bhaf005. doi: 10.1093/cercor/bhaf005 (PMC11795508; doi:10.1093/cercor/bhaf005)
Supplement: TMS_Szeged_Supplementary_material_final_bhaf005 [file tms_szeged_supplementary_material_final_bhaf005.docx]

# Enhancing Retrieval Capacity of the Predictive Brain through Dorsolateral Prefrontal Cortex Intervention

Running title: Enhancing Predictive Capacities via DLPFC

Laura Szücs-Bencze^1^, Teodóra Vékony^2,3,†^, Orsolya Pesthy^4,5^, Krisztián Kocsis^6^, Zsigmond Tamás Kincses^6^, Nikoletta Szabó^1,*^, Dezso Nemeth^2,3,7,†^

^1^Department of Neurology, Albert Szent-Györgyi Clinical Center, University of Szeged, Semmelweis utca 6, 6725 Szeged, Hungary

^2^Centre de Recherche en Neurosciences de Lyon CRNL U1028 UMR5292, INSERM, CNRS, Université Claude Bernard Lyon 1, 95 Boulevard Pinel, 69500 Bron, France

^3^Gran Canaria Cognitive Research Center, Department of Education and Psychology, University of Atlántico Medio, Ctra. de Quilmes, 37, 35017 Las Palmas de Gran Canaria, Spain

^4^Doctoral School of Psychology, ELTE Eötvös Loránd University, Kazinczy utca 23-27, 1075 Budapest, Hungary

^5^Institute of Psychology, ELTE Eötvös Loránd University, Izabella utca 46, 1064 Budapest, Hungary

^6^Department of Radiology, Albert Szent-Györgyi Clinical Center, University of Szeged, Semmelweis utca 6, 6725 Szeged, Hungary

^7^BML-NAP Research Group, Institute of Psychology, Eötvös Loránd University and Institute of Cognitive Neuroscience and Psychology, HUN-REN Research Centre for Natural Sciences, Damjanich utca 41, 1072 Budapest, Hungary

^*^Corresponding author: Department of Neurology, Albert Szent-Györgyi Clinical Center, University of Szeged, Semmelweis utca 6, 6725 Szeged, Hungary. E-mail: [szabo.nikoletta@med.u-szeged.hu](mailto:szabo.nikoletta@med.u-szeged.hu)

^†^These authors have contributed equally to this work.

**Supplementary Table 1.** Results of model comparison of the Learning session

| Model | Description | k | AIC | log-likelihood |
| --- | --- | --- | --- | --- |
| M1 | FE(Group × Block × Trial Type) + RE(1\|Subject) | 18 | 44951 | -22458 |
| **M2** | **FE(Group ×** **Block × Trial Type) + RE(Block\|Subject)** | 20 | **44497** | -22228 |
| M3 | FE(Block × Trial Type) + RE(1\|Subject) | 6 | 44992 | -22490 |
| M4 | FE(Block × Trial Type) + RE(Block\|Subject) | 8 | 44505 | -22244 |
| M5 | FE(Group × Trial Type) + RE(1\|Subject) | 10 | 46873 | -23426 |
| M6 | FE(Group × Trial Type) + RE(Block\|Subject) | 12 | 44671 | -22324 |

FE = fixed effects, RE = random effects, k = number of parameters, AIC = Akaike Information Criterion, BIC = Bayesian Information Criterion

**Supplementary Table 2.** Linear mixed model analysis of mean RTs of the Learning session

| Fixed effects | *b* | *SE b* | *95% CI* | *t* | *p* |
| --- | --- | --- | --- | --- | --- |
| (Intercept) | 359.62 | 3.97 | [351.83, 367.41] | 90.52 | < .001 |
| Bilateral DLPFC | 0.67 | 6.90 | [-12.86, 14.20] | 0.09 | .92 |
| Left DLPFC | 1.57 | 6.90 | [-11.95, 15.11] | 0.22 | .81 |
| Right DLPFC | 0.08 | 6.90 | [-13.44, -13.61] | 0.01 | .99 |
| Block | -13.26 | 0.73 | [-14.71, -11.81] | -17.93 | < .001 |
| Trial Type | -4.32 | 0.25 | [-4.83, -3.81] | -16.69 | < .001 |
| Bilateral DLPFC × Block | 1.91 | 1.28 | [-0.60, 4.43] | 1.49 | .13 |
| Left DLPFC × Block | -2.67 | 1.28 | [-5.19, -0.15] | -2.08 | .03 |
| Right DLPFC × Block | -0.77 | 1.28 | [-3.29, 1.74] | -0.60 | .54 |
| Bilateral DLPFC × Trial Type | 0.34 | 0.45 | [-0.53, 1.23] | 0.77 | .44 |
| Left DLPFC × Trial Type | -0.35 | 0.45 | [-1.23, 0.53] | -0.78 | .43 |
| Right DLPFC × Trial Type | 0.24 | 0.45 | [-0.63, 1.13] | 0.55 | .58 |
| Block × Trial Type | -1.50 | 0.25 | [-2.01, -0.99] | -5.79 | < .001 |
| Bilateral DLPFC × Block × Trial Type | 0.05 | 0.45 | [-0.82, 0.93] | 0.12 | .90 |
| Left DLPFC × Block × Trial Type | 0.34 | 0.45 | [-0.53, 1.22] | 0.76 | .44 |
| Right DLPFC × Block × Trial Type | -0.04 | 0.45 | [-0.92, 0.83] | -0.10 | .91 |
| Random effects |  |  |  |  |  |
| σ^2^ | 338.86 |  |  |  |  |
| τ00_Subject_ | 1586.93 |  |  |  |  |
| τ11_Subject.Block_ | 48.42 |  |  |  |  |
| ρ01_Subject_ | -0.38 |  |  |  |  |
| ICC | 0.83 |  |  |  |  |
| N_Subject_ | 101 |  |  |  |  |
| Observations | 5050 |  |  |  |  |
| Marginal R^2^/Conditional R^2^ | 0.93/0.84 |  |  |  |  |

**Supplementary Table 3.** Results of model comparison of the Retrieval session

| Model | Description | k | AIC | log-likelihood |
| --- | --- | --- | --- | --- |
| M1 | FE(Group × Block × Trial Type) + RE(1\|Subject) | 18 | 8455.6 | -4209.8 |
| M2 | FE(Group × Block × Trial Type) + RE(Block\|Subject) | 20 | 8419.2 | -4189.6 |
| M3 | FE(Block × Trial Type) + RE(1\|Subject) | 6 | 8477.2 | -4232.6 |
| M4 | FE(Block × Trial Type) + RE(Block\|Subject) | 8 | 8438.6 | -4211.3 |
| M5 | FE(Group × Trial Type) + RE(1\|Subject) | 10 | 8457.7 | -4218.9 |
| **M6** | **FE(Group × Trial Type) + RE(Block\|Subject)** | **12** | **8418.4** | -4197.2 |

FE = fixed effects, RE = random effects, k = number of parameters, AIC = Akaike Information Criterion, BIC = Bayesian Information Criterion

**Supplementary Table 4.** Linear mixed model analysis of mean RTs of the Retrieval session

| Fixed effects | *b* | *SE b* | *95% CI* | *t* | *p* |
| --- | --- | --- | --- | --- | --- |
| (Intercept) | 319.02 | 3.06 | [313.00, 325.04] | 104.03 | < .001 |
| Bilateral DLPFC | 5.34 | 5.32 | [-5.11, 15.80] | 1.00 | .31 |
| Left DLPFC | -3.51 | 5.32 | [-13.97, 6.93] | -0.66 | .50 |
| Right DLPFC | 0.71 | 5.32 | [-9.74, 11.17] | -0.13 | .89 |
| Trial Type | -5.41 | 0.38 | [-6.16, -4.66] | -14.11 | < .001 |
| Bilateral DLPFC × Trial Type | 2.01 | 0.66 | [0.70, 3.32] | 3.02 | .003 |
| Left DLPFC × Trial Type | -1.26 | 0.66 | [-2.57, 0.04] | -1.89 | .058 |
| Right DLPFC × Trial Type | -0.88 | 0.66 | [-2.19, 0.42] | -1.32 | .18 |
| Random effects |  |  |  |  |  |
| σ^2^ | 148.73 |  |  |  |  |
| τ00_Subject_ | 967.99 |  |  |  |  |
| τ11_Subject.Block_ | 19.88 |  |  |  |  |
| ρ01_Subject_ | -0.25 |  |  |  |  |
| ICC | 0.87 |  |  |  |  |
| N_Subject_ | 101 |  |  |  |  |
| Observations | 1010 |  |  |  |  |
| Marginal R^2^/Conditional R^2^ | 0.03/0.87 |  |  |  |  |


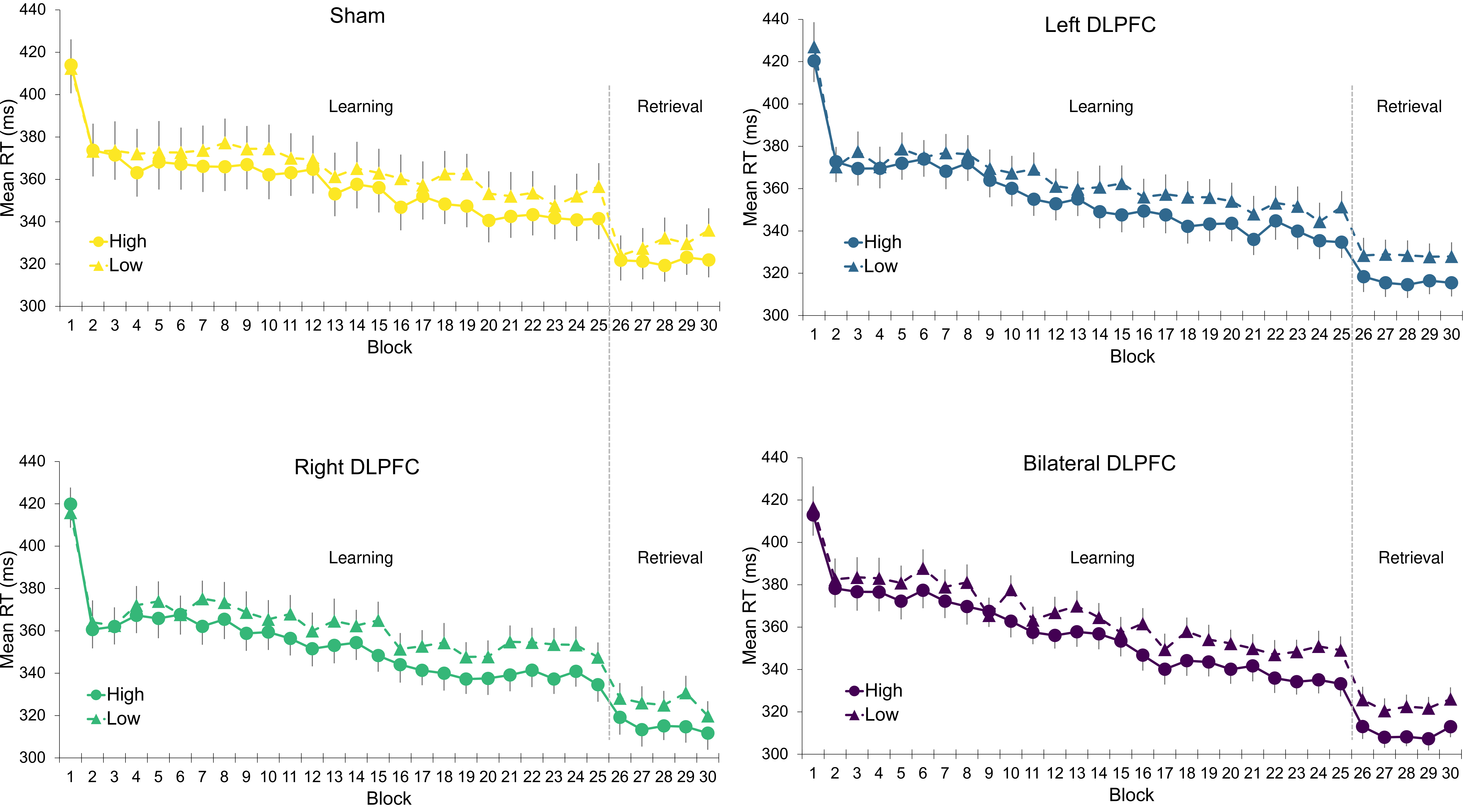


**Supplementary Figure 1.** Mean reaction times (RTs) of high-probability triplets (solid line with circle) and low-probability triplets (dashed line with triangle) are presented on the y-axis across all blocks (x-axis). A greater difference between the two triplet types indicates better statistical learning performance. Blocks 1-25 represent the Learning session, while Blocks 26-30 correspond to the Retrieval session. The performance of the four groups is shown with different colors. Error bars denote the standard error of the mean (SEM).
